# Lightweight Sensor Authentication Scheme for Energy Efficiency in Ubiquitous Computing Environments

**DOI:** 10.3390/s16122044

**Published:** 2016-12-01

**Authors:** Jaeseung Lee, Yunsick Sung, Jong Hyuk Park

**Affiliations:** 1 Department of Computer Science and Engineering, Soongsil University, Seoul 07027, Korea; ljs0322@ssu.ac.kr; 2 Faculty of Computer Engineering, Keimyung University, Daegu 42601, Korea; 3 Department of Computer Science and Engineering, Seoul National University of Science and Technology, Seoul 01811, Korea

**Keywords:** sensor network, sensor authentication, lightweight authentication, IoT authentication, IoT

## Abstract

The Internet of Things (IoT) is the intelligent technologies and services that mutually communicate information between humans and devices or between Internet-based devices. In IoT environments, various device information is collected from the user for intelligent technologies and services that control the devices. Recently, wireless sensor networks based on IoT environments are being used in sectors as diverse as medicine, the military, and commerce. Specifically, sensor techniques that collect relevant area data via mini-sensors after distributing smart dust in inaccessible areas like forests or military zones have been embraced as the future of information technology. IoT environments that utilize smart dust are composed of the sensor nodes that detect data using wireless sensors and transmit the detected data to middle nodes. Currently, since the sensors used in these environments are composed of mini-hardware, they have limited memory, processing power, and energy, and a variety of research that aims to make the best use of these limited resources is progressing. This paper proposes a method to utilize these resources while considering energy efficiency, and suggests lightweight mutual verification and key exchange methods based on a hash function that has no restrictions on operation quantity, velocity, and storage space. This study verifies the security and energy efficiency of this method through security analysis and function evaluation, comparing with existing approaches. The proposed method has great value in its applicability as a lightweight security technology for IoT environments.

## 1. Introduction

The technological paradigm that seeks to converge people, devices, communications, and data has evolved through the Internet of Things (IoT), Wireless Sensor Networks (WSNs), Ubiquitous Sensor Networks (USNs), Machine to Machine (M2M), and the Internet of Everything (IoE), and has most recently been established as Cyber Physical Systems (CPSs) [1]. These technologies are generally referred to as the IoT, and they use wireless communications technology to connect people with devices and devices with each other to provide smart technologies and services [2].

The various devices in the IoT environment process significant information; the IoT environment can analyze various aspects of the environment, economy, or infrastructure, and allocate devises depending on these results. Allocated devices can sense and process simple environmental information (such as temperature and humidity) or personal information (such as an individual’s location). In the case of processing simple environmental information, service quality is often prioritized over security, for the sake of efficiency [3,4]. However, even if the device only processes environmental information, services that involve smart energy or smart vehicles can be closely related to public and personal security [5,6].

As such, IoT devices must have security technology applied while accounting for the damage that could result from an intrusion, the device’s hardware functionality, the environment and infrastructure, and the type and importance of the information being processed. Many factors must be considered in IoT security environments, not limited to the characteristics of device communication in which data are transmitted through nearby nodes, the anticipation that more than 18 billion such devices will be in use by the year 2018, the vulnerabilities of existing security algorithms, the limits of security functions as defined in standard documents, and known vulnerabilities of authentication algorithms [7,8,9]. Thus, this study considers energy efficiency to make the best use of limited resources and suggests a lightweight mutual verification and key exchange method based on a hash function that has no restrictions on operation quantity, velocity, and storage space [10].

## 2. Related Work

### 2.1. Internet of Things

Internet-based intelligent device communication technologies provide mutual connection networks between heterogeneous smart devices, including mini-sensors. Thus, environmental characteristics, such as the power of the sensor and computer, memory storage space, battery capacity, and communication bandwidth should be considered in IoT environments. Lightweight Implementation Guidance (LWIG), from the Internet Engineering Task Force (IETF) standardization organization, classifies IoT devices from 0 to 2 based on resource restrictions [11]. Class 0 devices have less than 10 KiB memory, including super lightweight devices with 100 KiB of maximum load code capacity. Therefore, Class 0 devices, as designated by LWIG, should use access technologies such as IEEE 802.15.4 or Low Power, which is categorized as a Low Power Lossy Network (LLN), considering their cost and efficiency [12]. Heterogeneous devices, such as sensors or smart devices, that have different functions are being used in building automation, environment monitoring, energy management, and military purposes through network communication; technologies such as mutual verification between devices, message sending verification, and information confidentiality that compose the IoT environment should be provided. The current IETF CORE group is standardizing the Constrained Application Protocol (CoAP) for IoT environment [13,14], which applies security protocols used in existing Internet environments, such as Datagram Transport Layer Security (DTLS) and HIP, to provide secure services in resource-limited environments [15]. 

However, these systems are still flawed: in the case of DTLS, since the total six-message packet that is sent has the fate-sharing characteristic, the whole message should be re-sent if a packet is missing. Resending message packets can lead to network overload and the degradation of limited devices like mini-sensors [16]. Although security standardization is progressing for IoT environments, the lightweighting plan of suggested security protocols does not accept all super lightweighted devices in heterogeneous IoT environments [17].

### 2.2. Smart Dust

Smart dust originated with Kris Pister of the University of California, Berkeley, who developed a 1–2 mm mini-sensor. Though these sensors are very tiny (like dust), they possess significant computing ability, an electric power supply, bi-directional wireless communication, and a solar battery [18,19].

**Figure 1:**
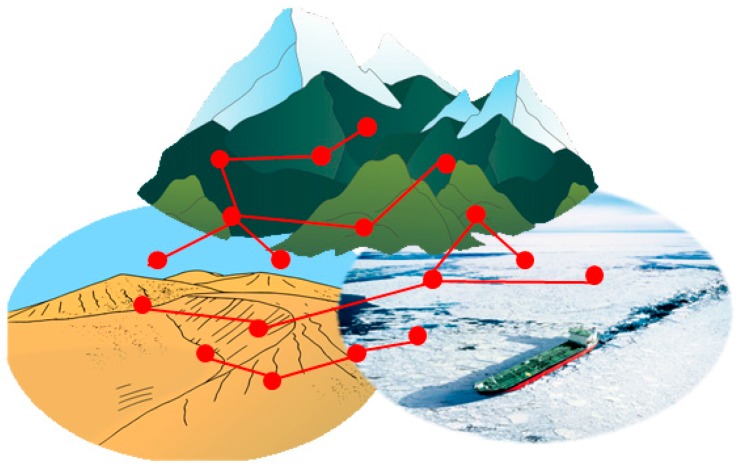
Smart dust utilization.

A smart dust network is shaped by multiple smart dusts, enabling not only mutual communication but also information collection. Management of areas such as military zones or inaccessible forested areas is a driving force in smart dust development. Although equipment such as unmanned surveillance drones and artificial satellites are currently used for surveillance, they risk exposure and their capacity for real-time information collection is limited [20,21]. Smart dust overcomes the limitation of their capacity by deploying sensor nodes to a variety of environments and collecting the real-time information as shown in Figure 1. dust-sized surveillance equipment distributed to necessary zones makes real-time information collection possible, which enables natural communication without environmental limitations. This equipment can also be tracked by recognizing its precise location and timing information. 

Like smart dust, the Internet was initially designed to serve military purposes; as the Internet’s sphere of application has been extended to daily life, the applications of smart dust are also evolving. Intel and UC Berkeley built a wireless network using smart dust that remotely monitors the condition of sea swallows. The technology is also being used in to prevent cold-weather damage of crops, make ecological observations in inaccessible areas, measure biochemical pollution, prevent forest fires, observe the weather and earthquakes, detect the movement of troops and equipment, and manage distribution. For example, smart dust that prevents crop damage from cold weather may collect soil information in real time by planting smart dust in the soil. In the Telecommunication Engineering laboratory of Twente University in the Netherlands, smart dust distributed in mountains form a wireless network that is used to conduct smart dust research with real-time information delivery to a monitoring center [22]. 

Potential applications of smart dust are inexhaustible; although the technology is currently highly utilized, its use is expected to spread as it is combined with a variety of additional technologies [23].

### 2.3. Previous Sensor Technologies

#### 2.3.1. Blundo Scheme

The Blundo protocol [24] is a method in which each node in a network generates a common key in a group environment that has *t* nodes. First, it selects a symmetric polynomial that is *P*(*x_*1, *…*, *x_t*) with degree *k* on *t* variable units at the server, and gives each member a symmetric polynomial, *f*(*x_*1, *…*, *x_t*). *f*(*i*) is substitute *i* for *x* in *P*(*x_*1, *…*, *x_t*). *User_*(*ji*), *…*, *User_jt* (members who received the polynomial) calculate with their IDs to obtain *s_*(*ji*, *…*, *jt*) which is identical in value to *P*(*j_*1, *…*, *j_t*). Although this method is secure as long as *t* member units are not attacked, many calculations are demanded of each node in the sensor network.

#### 2.3.2. Blom Scheme

If *a* and *b* want to communicate, each first changes the selected row vector from its matrix and multiplies the opponent’s row vector by its line vector. When we call matrix *K GT* × *D* × *G*, *a* gains matrix *K*’*s* (*j*, *i*) entry and *b* gains the (*j*, *i*) entry from the above calculation. Since *K* is a symmetric matrix, both entries have the same value; thus, a shared key between nodes *a* and *b* may be found. In this method, if the number of damaged nodes is less than λ, security provides assured; however, if the number more than λ nodes are damaged, all secure information can be exposed. This limitation of the Blom method is called λ-security [25].

#### 2.3.3. PCGR

**Figure 2:**
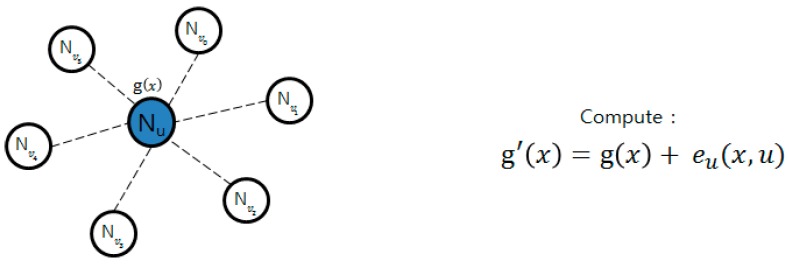
Group key generation and encryption.

**Figure 3:**
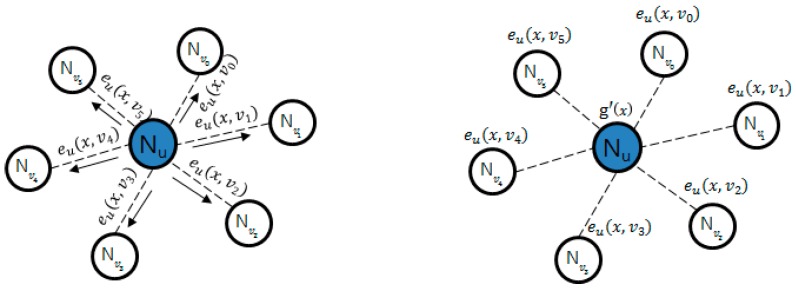
Group key distribution.

**Figure 4:**
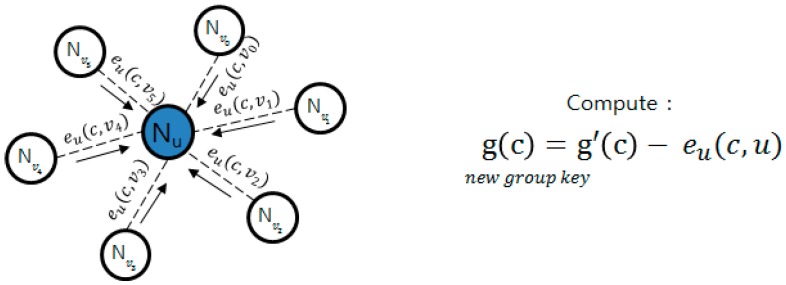
Group key update method.

In of the method described by Zhang [26], key information is pre-distributed and group key is regenerated through cooperation between the nodes. This technique prevents the group key that will be generated next from being known in case a group-key-making node is attacked. In Figure 2, the group key is generated through *g*(*x*); as it generates *e*(*x*, *y*), it encrypts *g*(*x*) as follows. After encryption are transmitted by the form, *e*(*x*, *y*), *e_u* (*x*, *v_i*), to around nodes as shown in Figure 3, while g’(x) owned by it self is not deleted, g(x) and e(x) are deleted. When the key is renewed, the time managed by each node is used, and when time is expired, pieces that each node requires for renewal are sent, as in Figure 4. Nodes that sent pieces get *t +* 1 of *e_u* (*c*, *v_i*) pieces returned from the node and the group key is renewed with the following calculation. Here, *c* points out the group key version using count value.

Even if nodes are attacked, the next group key is not revealed, through the node that received the group key with the general node’s calculations cannot prove whether the group key is correct.

#### 2.3.4. SLIMCAST

Huang suggested the technique of re-encrypting data in a gradational key structure to provide data confidentiality at each hop-by-hop pass [27]. A clustered group is divided into routing trees and encrypts communication between the nodes with the key at the same level; the key is renewed when a node enters or leaves the network. In the active leaving case, a node directly informs the cluster header of its departure. In the passive leaving case, nodes are removed without informing the cluster header because of hardware problems or physical damage. Finally, in the case of deleting malicious nodes, nodes with abnormal behavior are detected by the base station, which informs the cluster header to remove the node from the group. SLIMCAST has good overhead and energy efficiency, but its working speed decreases if many nodes are changed.

### 2.4. Previous Crypto Technology

The RSA algorithm, which is widely used for the safe distribution of encryption keys and other key management problems, is an encryption method based on the theory that generally, multiplying two prime numbers with a large difference between them is easy, but factoring the two numbers once multiplied is difficult [28]. However, the algorithm places a large operational demand on devices during the encryption and decoding processes; if it were applied in IoT environments, the network may become overloaded.

Elliptic curve cryptosystems (ECCs), based on the discrete logarithm in the elliptic curve group defined in finite fields, is a public key encryption algorithm suggested independently by Miller and Koblitz in 1985. It has been studied in number theory and algebraic geometry for over 150 years. In recent encryption research, the elliptic curve method (ECM) has been utilized in factorization problems, primality tests, and public key encryption, which are crucial for RSA encryption [29]. 

Currently, public key encryption utilizes intractability; that is, the calculation is possible, but takes significant time to calculate, according to computational complexity theory. The early stages of public key encryption are based on prime factorization with random positive integers, which requires significant time to perform. Elliptic curve encryption relies on the long time needed to find the discrete log of a random elliptic curve at a specific known point. For encryption purposes, an elliptic curve is a type of plane curve and is the set of points that meet the equation *y*^2^ = *x*^3^ + *ax* + *b.* (To simplify the curve, the point characteristics are fixed finite fields). The above set forms the Avenlan group, having the infinite point as the identity element together with the computation of the elliptic curve group. The structure of the group follows the factor of the underlying algebraic variety [30,31].

Kerberos authentication is composed of a Kerberos server, ticket, and authenticator. After the Kerberos server and Ticket Granting Server (TGS) generate a ticket, the ticket is used in communication between the client and TGS, and between TGS and the server. The authenticator is generated by the client, can be used only once and includes the client name, workstation IP address, and the current time as authentication information [32]. When the client request a login to request a server access ticket, and authentication is completed by transmitting the required information, the ticket is generated and sent to the client; then it verifies whether the client is authorized from the server. The user saves the ticket and accesses TGS using this ticket when requesting access to the service. To prevent ticket interception, it includes the ticket issuance time and valid time. The AS verifies the ID by providing the session key between client and TGS and between client and server, and prevents the risk of intercept by limiting the valid time [33]. Despite this, intercept and replay attacks are possible using the weak point in that time. Although user authentication is realized by issuing the ticket, a digital signature is not provided. In addition, due to security problems in the Kerberos server, it should be constructed with perfect confidence between client and server, as well as between servers. That is, it should be used with encryption technology such as SSL [34] or Diffie-Hellman [35], though these can cause network overloading when applied to IoT environments [36].

## 3. Proposed Scheme

In the suggested method, while routing protocol motion is based on the LEACH routing protocol [37], a lightweight protocol for existing sensor network environments, it forms a group using energy information for greater efficiency. The suggested IoT environment has the following characteristics: every sensor node in the specific area can transmit to the sync node in one hop; sensors give the intensity of received power and changes in transmission power are possible; and sensor nodes regularly transmit data to the base station.

For the battery life, each sensor periodically selects a random Middle Node (MN) and forms a group through it. After the sensor nodes belonging to the specific group collect data to transmit to MN, MN aggregates this information and transmits it to MD. At this time, MN consumes relatively more energy compared to other nodes and may negatively influence the whole network if specific nodes become overloaded. Thus, after a specified time period, MN is selected in order to distribute its energy consumption by round. 

At this time, to consider the energy efficiency of sensor nodes, nodes belonging to each group transmit their energy quantity and MN aggregates this information and transmits it to MD. MD selects the top 30% nodes that do not play a role in MN through a probability algorithm, as follows:

(1) $$P_{i}\left(x\right)=\frac{K_{opt}}{N-K_{opt}\left(r\;mod\;\frac{N}{K_{opt}}\right)}C_{i}\left(t\right)=0\;or\;1$$

where *i* represents each sensor and has a value ranging from 1 to the total number of sensors, *N*; *t* is time; *P_i_*(*t*) is the probability of selecting *i* as MN at *t*; *r* represents the round; *K_opt_* is the number of rounds that ends at Group of Round; and *C_i_*(*t*) represents either 0 or 1, according to whether it was selected as MN before *t*. One round is performed from when a new cluster is formed to when the new cluster is expired. When all nodes have each cluster head, the nodes can be eligible to become cluster heads, which state is defined as Group of Round. As the round progresses, since the number of sensors participating in MN selection decreases, the probability of becoming MN increases. At this point, the probability of becoming MN is *K_opt_*.

The sensor that calculated *P_i_*(*t*) will pick a *p* value between 0 and 1; if *p* < *P_i_*(*t*), it selects itself as MN. If *p* > *P_i_*(*t*), the node will be used for forming a cluster. If the current node became MN at Group-of-Round, *P_i_*(*t*) = 0, so it is impossible to become MN until a new round begins. In the final round, *P_i_*(*t*) = 1, so nodes that never have been MN are selected as MN. The parameters for the proposed protocol are described in Table 1.

### 3.1. Initial Authentication Process

**Figure 5:**
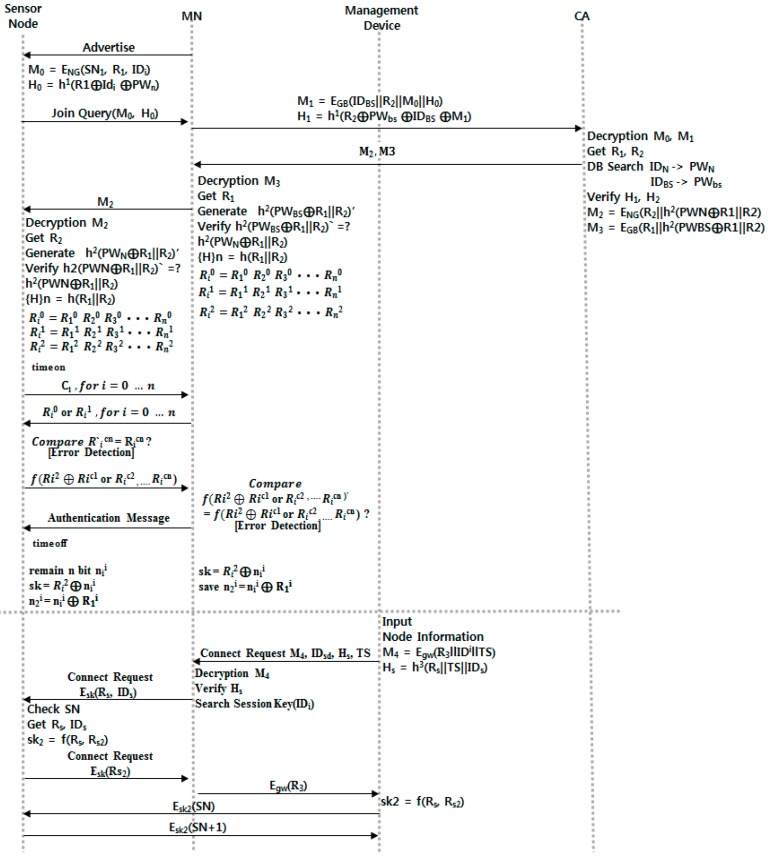
Initial authentication and device registration process.

Figure 5 shows Initial Authentication Process and Smart Device Registration. The gateway that controls the home network of a specific area constantly sends advertisement messages. Smart nodes that are newly registered in the network system send join messages in response to these advertisement messages; the gateway verifies smart nodes through CA. When verification is completed, a smart device carries out mutual verification and key exchange as follows [38].

**Step** **1.** The smart node that received an advertisement message generates *M*_0_ and *H*_0_ for self-verification and sends them to the gateway.
**Step** **2.** The gateway that received *M*_0_ and *H*_0_ generates *M*_1_ and *H*_1_ and sends them to the CA manufacturer.
**Step** **3.** The CA that received information from the gateway obtains two random numbers through decoding and verifies *H*_0_ and H_1_ through searched *PW*. It generates *M*_2_ and *M*_3_ and sends them to the gateway.
**Step** **4.** The gateway that received *M*_2_ and *M*_2_ gains *R*_1_ from decoding *M*_3_ and checks for any error in the received value through a hash function. It saves 3 × *n*-bit information generated by two random numbers for verification and then sends *M*_2_ to the smart node.
**Step** **5.** The smart node that received *M*_2_ gains *R*_2_ and verifies the sent value through a hash function. If the verification is finished, the node saves 3 × *n*-bit information, as above.
**Step** **6.** The smart node generates random number *C*_1_ to perform the verification step and sends it by bit. At this point, the time check for preventing relay attacks begins.
**Step** **7.** The gateway that received bits from the smart node sends the *i*th bit of *R*_0_ if *C*_1_ = 0 in response, and sends the *i*th bit of *R*_1_ to the smart node if the response is 1.
**Step** **8.** The smart node generates *R_i_^cn^* based on the *c* sent to the gateway and compares it to *R_i_^cn^*, which is the gathered value of the cluster head’s response to verify whether the data was sent from the correct node. After “time off,” it guesses the distance through time measurement and stops communication if this is greater than a specified time.
**Step** **9.** The smart node that verified the gateway sends the received *R_i_^cn^* values using the *f*() function to the gateway.
**Step** **10.** The smart node that received a certification value from the gateway generates a certification value in same way and compares it to the value received from the cluster head to verify the smart node.
**Step** **11.** The smart node and the gateway generate a session key using the left *n* bit and a random number from the 3 × *n*-bits and ends the verification.

### 3.2. Smart Device Registration

**Step** **1.** A user who tries to control the smart node through a smart device generates a random number and M4 and Hs, and then sends M4, IDsd, Hs, and TS to the gateway.
**Step** **2.** The gateway that received this decodes M4 and verifies Hs. After that, it searches the session key with the smart nodes and sends the encrypted Rs and IDs.
**Step** **3.** The smart node generates session keys through Rs; after it generates R3, it sends the encrypted session key with the gateway. The gateway sends them through the key with the smart node after decoding.
**Step** **4.** The smart device generates a session key with two random numbers and ends the procedure after verifying them through the certification value that uses the smart node and serial number.

### 3.3. Smart Device Key Updating Process

**Figure 6:**
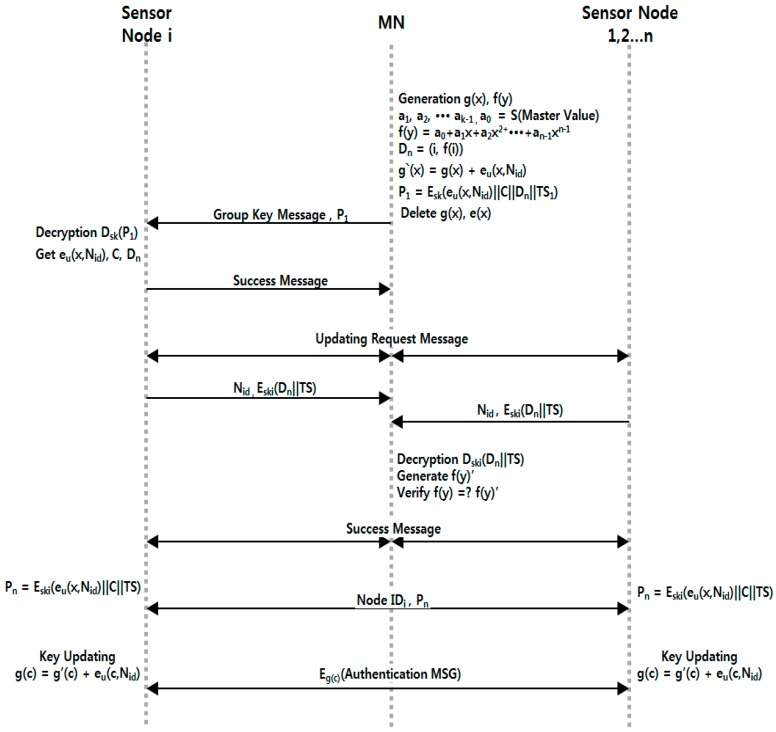
Group key updating process.

**Step** **1.** After completing certification sensor group formation and session key distribution, the sensor node carries out the group key distribution and renewal process as follows. Using the polynomial distribution of PCGR, it makes MN do most of the calculation, distributes pieces of the specific value through the *f*(*y*) function, and verifies whether the node is contaminated or needs to be withdrawn.
**Step** **2.** The group’s representative node, MN, defines and generates polynomials *g*(*x*) and *f*(*y*) for group distribution and node verification. After that, it generates verification values S and *D_n_*. Next, it generates *P*_1_ for transmitting to nodes and deletes *g*(*x*) and *e*(*x*) to prevent them from being exposed by an attacker.
**Step** **3.** The sensor node that received P_1_ decodes this, and obtains the group key and secret piece. It then informs MN that the group key was successfully received.
**Step** **4.** After a certain period of time, MN transmits a message for updating the group key to the nodes within the group. Nodes that receive the key-update message send MN an encrypted secret piece with the session key in response, and MN checks the validity of the received value through a Lagrangian polynomial and sends a “success” message. If the value differs, it informs specific nodes of contamination.
**Step** **5.** Nodes that received the message from MN send nodes around *P_n_* for updating the key through Equation (2):
  (2) $$g\left(c\right)=g\prime \left(c\right)+e_{u}\left(c,{\;N}_{\mathrm{id}}\right)$$
**Step** **6.** After the key is updated, it finishes the verification process with nodes through the group key. The whole process of Smart Device Key Updating is shown in Figure 6.

## 4. Performance Evaluation

### 4.1. Securiy Analysis 

#### 4.1.1. Mutual Authentication 

This paper uses random numbers generated by the smart nodes and gateways when bits are exchanged to generate *R_i_°*, *R_i_°* and *R_i_°*, *R_i_°*, which are promised as responses to the random value *c*, making mutual authentication possible. In addition, for the registration of smart devices, authentication is possible through the authenticated gateway. 

#### 4.1.2. Replay and Relay Attacks

This is a method of attack in which a message is stolen by an unauthenticated attacker while the message is sent to each node. When the message is reused, a newly generated session key and not the session key that occurred during the steal, *sk* = *R_i_°* ⊕ *n*_1_ and *f*(*R_s_*_1_, *R_s_*_2_), is used. This makes it secure against attacks where previous messages are reused. For each message, time stamps are generally applied. Verification is possible through the completion of the message time. Through the bit exchange process, it can be judged whether each node is physically close, making it secure against relay attacks. 

#### 4.1.3. Message Manipulation Attacks

This is an attack in which an unauthenticated attacker steals the message transmitted to each node and makes it a counterfeit message to meet the goal of the attacker. In this case, too, a newly generated session key and not the session key that had been generated at the time the message was sent, *sk* = *R_i_°* ⊕ *n*_1_ and *f*(*R_s_*_1_, *R_s_*_2_), is used. This makes it secure against counterfeit message attacks. 

#### 4.1.4. Snooping

This is an attack method in which the transmitted message is snooped. Since it uses *f*(*R_s_*_1_, *R_s_*_2_) between the smart device and smart node, which is constantly updated, even if message snooping is attempted, only the encrypted phrase can be seen. 

#### 4.1.5. Spoofing

This is an attack in which network identification information is changed to deceive another party using previously authenticated nodes. Even if there is a spoofing attack, a session cannot be established through a third party, making the network secure against such attacks. 

#### 4.1.6. Side Channel Attack 

This is an attack in which side elements such as processing time, energy usage, or electronic waves are used. Regardless of the length of the data transmitted, the same size message is sent, making it secure against side channel attacks. 

#### 4.1.7. Forward Security and Error Detection

The sensor nodes MN and GW know where the bit that is generated through the bit stream calculation is located among the transmitted 2 bits. Through this, even if one bit is incorrectly located, error detection is possible and forward security can be achieved.

### 4.2. Overhead Analysys

Blundo’s protocol, which is the sensing security technique in existing sensor networks, differs depending on the number of nodes in the network; if the number of nodes is *N*, polynomial expression with (*N* − 1) variables of degree *t* are calculated. Each sensor node should save the polynomial expression of length *N*(*t* + 1)*L* in the server, requiring *N* messages. That is, Blundo’s Protocol can be secured unless *t* members are attacked, but it can overload the network, as each node performs significant calculation for an IoT environment in which numerous sensors exist. Blundo’s protocol provides good security when the number of damaged nodes is less than λ; if the number of damaged nodes is greater than λ, all secure information is exposed to danger. In the case of the PCGR technique, information to be saved and calculations required vary depending on the surrounding nodes generated the group key. Assuming that the number of surrounding nodes is *n* and the probability that the surrounding nodes generate the group key is *Pro*(*d*), it has a polynomial expression of the group key encrypted with (*t* + 1)*L*-bit length and bit information of *n*(*t* + 1)*L*, which is partial information of the surrounding nodes. In addition, for the message, aside from *n* messages, *Pro*(*d*) × *n*^2^ additional messages are needed. That is, for PCGR, if the cluster header, which distributes the group key within the group, is increased, energy consumption increases drastically. The more the nodes within the group increase, the more the average energy consumption is increased as well. In the suggested scheme, through the exchange in small bit units and hash function-based operation, few calculations are required within the group.

### 4.3. Analysis of Energy Eefficiency

The following simulation was performed using the MATLAB program in order to analyze the efficiency through the measurement of time efficiency for each IoT device for proposed protocol and the previous encryption technology.

Table 2 is the initial setting value for performing the simulation and the time for simulation was set considering the amount of node energy. For simulation, the time efficiency was measured according to the increase in IoT devices by setting a total of 10~200 nodes, and the test was performed by configuring an independent environment for each encryption technology and the proposed scheme. 

The sensor nodes were distributed randomly in a 20 m × 20 m area regardless of the number of nodes. The gate was assigned in a specific position considering the placement area. 

According to the placement, the distance between all sensor nodes and gateway remained between 25 m and 10 m. Each round was set to last for 20 s.

**Figure 7:**
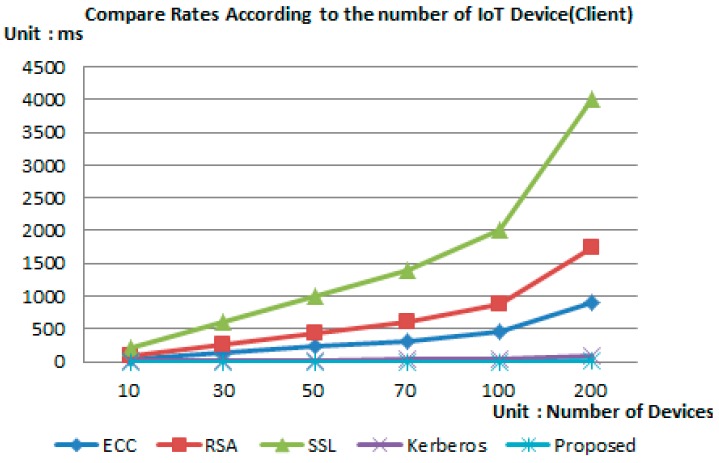
Analysis of authentication time—client.

**Figure 8:**
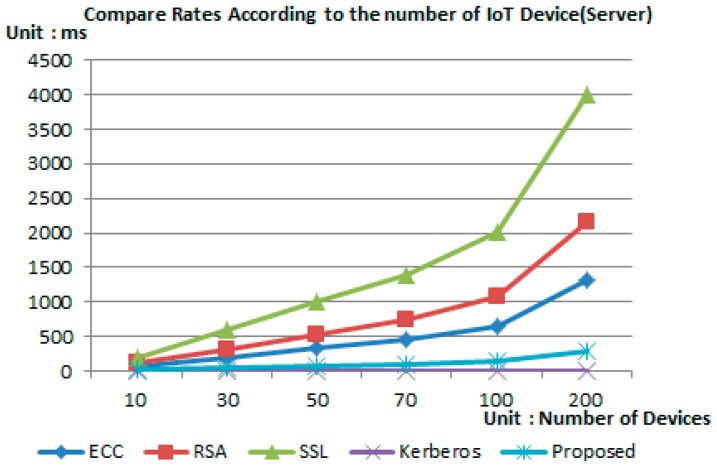
Analysis of authentication time—server.

Figure 7 and Figure 8 show the simulation result after the environment was configured as shown in Table 2. Simulation results have shown that in the case of the proposed scheme, the operation being performed in the device was relying on simple hash operation and arithmetic operation with less operation consumption time compared to other authentication techniques. 

In addition, in the case of group key distribution and renewal process, all polynomial operation was performed by the server, thereby reducing the burden of the device and providing a lightweight characteristic compared to other authentication techniques.

## 5. Conclusions

The proposed scheme uses simple hash calculations to support devices with hardware limitations, and suggested certification protocols to focus on specific nodes for complex calculations. We consider a routing protocol plan in existing sensor network environments for the commercialization of certification protocols that was designed to accept super-lightweight devices that cannot equip encrypted modules in a heterogeneous IoT environment, a limitation of existing IoT environments.

Recently, research related to efficient lightweight certification and key management approaches such as Danyang’s Protocol [39] and Thaier’s approach [40] has been introduced. However, given that WSN environments are extended to the concepts of the IoT, there is a limitation in that the security requirements and environmental elements defined in IoT standards are not satisfied. The proposed scheme designs the protocols considering not only the environmental elements of OneM2M but also the environmental elements of smart dust where the smart dust is expected to be one of the major technologies.

We confirmed through performance analysis that our certification framework is more lightweight than renowned security certification techniques, and largely improved in security and energy efficiency with less energy consumption compared to existing certification techniques. Security comparison analysis shows that the method meets every security requirement defined by the OneM2M standard. Thus, as the lightweight certification and key management scheme in the suggested IoT environments meets the OneM2M international security standards, we confirm that it is superior to existing security techniques. 

Due to the hardware limitations of subminiature devices, continuous group renewal may efficiently deal with addition and withdrawal of new nodes. As its security was verified, the proposed method is applicable for various environments in which a variety of devices, including simple subminiature sensors, are used; it is also expected to be practical for IoT environments.

## Figures and Tables

**Table 1:** Proposed protocol parameters.

Notation	Meaning
*N_v_*, *N_p_*	Nonce
*MN*	Middle Node
*CA*	Certificate Authority
*ID*	Node ID
*C_id_*	Middle Node ID
*R_i_*°, *R_i_*^1^, *R_i_*^2^	3n Bit Divided Value
*Sk*	Session Key
*Nk*	*f*() Function Shared Key
*N_i_*	After distance bounding remaining bits
*C_i_*	Random bit
*g*(*x*)	Group Key Polynomial
*e*(*x*, *y*)	Group Key Encryption Polynomial
*g*’(*x*)	Encrypted Polynomial to *e*(*x*, *y*)
*E*(), *D*()	Encryption, Decryption

**Table 2:** Initial simulation setup table.

Initial Set Value for Simulation	
Number of sensor node	10~200
Placement area of the sensor	20 m × 20 m
Position of the gateway	*x* = 25 m, *y* = 10 m
Node initial energy	1.0
ETX, ERX	25 nanoJ
Eamp	50 picoJ
EDC	5 nanoJ
Packet size	2500 bit
Compressibility	0.05

## Abbreviations

The following abbreviations are used in this manuscript:

IoT	Internet of Things
WSN	Wireless Sensor Network
USN	Ubiquitous Sensor Network
M2M	Machine to Machine
IoE	Internet of Everything
CPS	Cyber Physical Systems
LWIG	Light-Weight Implementation Guidance
IETF	Internet Engineering Task Force
LLN	Low Power Lossy Network
CoAP	Constrained Application Protocol
DTLS	Datagram Transport Layer Security
ECC	Elliptic Curve Cryptosystem
ECM	Elliptic Curve Method
